# Uncovering inequities: the role of epidemiology in confronting racism

**DOI:** 10.1590/1980-549720250008.supl.1

**Published:** 2026-07-24

**Authors:** Joilda Silva Nery

**Affiliations:** I Universidade Federal da Bahia, Institute of Public Health – Salvador (BA), Brazil.

**Keywords:** Racism, Epidemiology, Racial inequalities, Black population health, Collective health

## Abstract

This article discusses the role of epidemiology and collective health in addressing racism and racial health inequalities in Brazil. The reflections presented were inspired by the conference “Unveiling Inequities: the role of epidemiology in confronting racism”, held during the 12th Brazilian Congress of Epidemiology in 2024. Through a critical and historical analysis, the study seeks to understand how the Brazilian field of epidemiology – consolidated in Latin America and recognized for its contributions to collective health – still reproduces mechanisms that render Black and Indigenous researchers invisible. It argues that combating racism should not be the isolated responsibility of a specific group, but rather a collective and ethical commitment involving the entire scientific community. Morbidity and mortality data speak for themselves and for the majority of the Brazilian population, revealing the urgent need for a more explicit debate on racism within epidemiology and for reflection on how institutions and research groups are organized and operate based on power relations. Furthermore, it proposes that contemporary topics such as digital health, climate change, and mental health be analyzed through intersectional perspectives that consider race, gender, class, and other dimensions of inequality. The text emphasizes that recognizing racism as a social determinant of health is an essential condition for building an epidemiology committed to equity and the right to life. Acknowledging these gaps is necessary to move forward, as highlighted in Brazil’s Fifth Brazilian Plan for the Development of Epidemiology.

This writing originates from reflections shared at the conference titled “Unveiling Inequities: The Role of Epidemiology in Confronting Racism,” held at the 12th Brazilian Congress of Epidemiology in 2024, and addressed the role of epidemiology in tackling racial health inequities in Brazil^1^. The text offers a critical analysis of epidemiology’s ethical and political commitment in the face of racial health injustices, problematizing how the field is structured, how it produces knowledge, and how it relates to populations affected by racism.

This trajectory has been essential for understanding how a diversity of “knowledges and practices” can strengthen epidemiological knowledge produced in universities and research institutions. To that end, we must first question with whom, for whom, and how we engage in the field. How can collective health and epidemiology contribute to effectively confronting racism? This requires a genuine commitment from the majority of people who make up the field. Many meetings, convergences, disappointments, affections, conflicts, lessons, and exchanges among peers lead me to reflect on our responsibility and engagement as epidemiologists in this political agenda.

Latin American and Brazilian epidemiology is an established field, but despite its undeniable contribution to population health, it is marked by the invisibilization of Black researchers who contributed to its history. Often, when the history of epidemiology is told, the movements of Black women who worked within the scope of the Brazilian Sanitary Reform are not taken into account. Authors such as Alaerte Coutinho, Maria Inês Barbosa, Edna Araújo, Denize Ribeiro, Lúcia Xavier, Jurema Werneck, and Fernanda Lopes have helped to situate racism as a social determinant of health and to demand that the topic be included on academic and political agendas. However, their contributions are still little recognized in curricula and in the historiography of Brazilian epidemiology. Fátima de Oliveira, in her contribution to Sueli Carneiro’s thesis^2^, urges us to consider that invisibilization is an old and successful racist political strategy.

As Chimamanda Ngozi Adichie^3^ warns us, there is the danger of the single story told by those in positions of privilege in the field, since many Black intellectuals do not appear. As our elder leaders teach us, we have history, memory, and we must preserve them. In this text I engage with intellectuals from different regions, backgrounds, and generations who have contributed day in and day out and over decades to confronting racism by producing evidence of how ethnic-racial inequities unevenly affect the birth, illness, and death^4^ of Black and Indigenous people in Latin America and Brazil. These people have made an enormous contribution to public health and epidemiology and should be cited and regarded as references.

Despite the hard-won advances, we Black, brown, and Indigenous people want and can make much more progress in strengthening public health. Sociodemographic and epidemiological data from different sources point to the worst living and health conditions for Black people worldwide and in Brazil. Some of the data needed to carry out health situation analyses are available in articles, book chapters, informational panels, and debates. I ask permission to speak for the thousands of people who make up the data that are analyzed and discussed in classes, research, and in supporting decision-making in Brazil’s Unified Health System (SUS).

The Black population (composed of people who identify as pardo and preto in Brazil) is not homogeneous and constitutes a significant share of traditional communities, quilombola and riverside communities; artisanal fishermen; people experiencing homelessness; people deprived of liberty; those living in extreme poverty and in dwellings that do not meet habitability standards, lacking water supply and/or sanitation as in favelas; those with the lowest incomes or who survive through informal work; people who depend on recyclable (or nonrecyclable) waste; domestic workers; older adults; those facing food insecurity; and those who have difficulty accessing health care, social assistance, and educational services and facilities^5,6^. We are talking about people who experience racism and various forms of violence in their daily lives, with significant repercussions for their subjectivity and their mental and physical health. How can we not be outraged?

The position people occupy in society results from historical processes and from the political, economic, and social conditions that shape inequalities produced by race, gender, generation, and social class. With regard to racial relations, it is important to remember that the term “race” was, for a long time, used to rank populations, supporting concepts of racial supremacy and biological purity. As Lopes and Werneck argue, the concept of race is not formed neutrally: it is structured by racism itself, understood as a social mechanism of exclusion that affects everyone, though in different ways depending on each group’s position in the social hierarchy^7^.

Dialogue with colleagues and readings from texts across different fields reinforce that epidemiology plays a fundamental role in revealing social and racial health disparities, but more progress is needed. Epidemiology, as a field of public health grounded in evidence production and guiding health decision-making, must consider addressing racial inequities as a priority strategy to improve morbidity and mortality indicators in Brazil; it must assume racism as central to the social determination of health inequalities. With racism, there is no health^7^, and epidemiology should identify priorities and paths to overcome these inequities.

Racism, as an ideological and structuring phenomenon, comprises a set of attitudes based on racial prejudice, discriminatory behaviors, institutional mechanisms, and social dispositions that assign negative value to diversity and produce hierarchical meanings among social groups. These dynamics sustain patterns of exclusion that result in persistent racial inequalities. Racism, therefore, shapes forms of coexistence, regulates social relations, and influences the organization and functioning of institutions. In the case of the Black population, the inequalities in being born, living, falling ill, and dying are neither natural nor inevitable – they are the product of a social system that could be transformed if racism were widely recognized as a social determinant of health and its confrontation assumed as an ethical and political priority both within and outside the services that make up SUS^4,8,9^.

How do non-Black epidemiologist researchers view the *pardo, preto*, and Indigenous people who make up the “samples” in their studies? Many published epidemiological studies highlight a “race cut,” a “gender cut” (often using sex categorized as male and female), or a “race/gender cut.” This perspective is often established by white men who do not see themselves as members of a group that is also racialized, but who do not experience racism. In doing so, they carry out epidemiological analyses using a racial breakdown that refers to *pardo, preto*, and Indigenous populations. According to the latest census from the Brazilian Institute of Geography and Statistics (IBGE)^10^, the Black population (*pardos and pretos*) represents 55.5% – that is, the majority of the Brazilian population. In this light, it is conceptually wrong to treat studies that address racial issues as merely a “cut”.

Chronic and infectious diseases; work-related accidents; hazardous and/or unhealthy occupations such as general services, cleaning, agriculture, and construction. Which ethnic-racial groups are most affected or die most from these diseases/accidents? What race/color, gender, and age groups make up the majority of these workers? In this sense, greater ethnic-racial detail on work-related diseases and injuries in Brazil is also necessary^11^.

Discussions with colleagues in public health indicate that racism is also repeatedly practiced by political actors operating within institutions and even in more progressive spaces (those who shape narratives in health and epidemiology but still refuse to recognize its depth as a cause of illness and death that victimizes Black and Indigenous populations). Academic works and events in the field are predominantly framed around class inequalities; race/color data are collected, presented, and discussed without sufficient analysis of how racism is a fundamental social determinant of health for understanding the exposures and outcomes under investigation. The gap in most epidemiological studies is the failure to recognize that the sociodemographic variable race/color is shaped by racism, which must be considered when interpreting the inequalities this variable reveals. Thus, confronting racism through research, teaching, and outreach in epidemiology should not be left to the few Black and Indigenous researchers who experience its daily dimensions. It is necessary to more explicitly debate racism within epidemiology and reflect on how our institutions, research groups, and thematic collectives are organized and operate in their power relations.

The racial issue in Brazil is not an identity matter. It is as structural and cross-cutting as class inequalities and, given our country’s historical formation, must not be disregarded. The data speak for themselves and for the majority of the Brazilian population. Discussions on the guidelines of the 5th Master Plan for the Development of Epidemiology of the Brazilian Association of Collective Health (Abrasco)^12^, considered inequalities/inequities in health, especially regarding the populations most affected by diseases and injuries; however, they still do not address the racial issue in a cross-cutting way, favoring “social inequalities”. While theoretical frameworks on class inequalities are necessary and important, they do not always adequately account for discriminations related to race, ethnicity, gender, and generation, highlighting the need to broaden the perspective on the structural issues affecting population health. The starting point for an epidemiology that reflects Brazil’s diversity lies in the genuine understanding that each quantitative datum represents lives and histories.

In health surveillance, ethnic-racial discussion focused on Black and Indigenous populations is still nascent in Brazil. The race/color variable is often treated merely as one item among many sociodemographic variables. Most publications on infectious diseases affecting Black people come from qualitative research, while the few existing epidemiological studies do not sufficiently analyze racism as a structuring factor in the social determination of health-disease processes. Much of the discussion about communicable diseases, both in epidemiology and clinical practice, centers on the diseases themselves, rendering invisible the people affected and those who experience the worst treatment outcomes when care is available^11^.

Repeating these epidemiological data impartially and uncritically, without probing the underlying causes, creates a discomfort similar to history classes about Brazil in schools. In those classes, the trajectories of Indigenous and Black populations were presented exclusively from the perspective of invisibility and enslavement. Collecting, analyzing, and systematizing epidemiological data that show the Black population has the worst morbidity and mortality indicators, while framing the debate solely in terms of class inequalities, constitutes a way of narrating the history of “the others”^3^.

In teaching and research institutions that have epidemiology departments, the intersections of race, ethnicity, gender, and generation are still systematically neglected. Despite the increase in publications using race/color as a variable, epidemiological studies that deepen theoretical and methodological discussions about the impacts of racism on health through an intersectional lens remain scarce. In this context, I refer to Phyllis A. Jones, who warns us about institutionalized racism as an evident inaction in the face of need^13^. We need to extend epidemiological discussion to ethnic-racial inequalities, but failure to do so contributes to the maintenance and perpetuation of racism.

Racism is part of the social determination of health, and the institutional dimension affects power relations within groups and institutions as well as the ways data, information, and knowledge are produced in epidemiology. First, we need to engage in reflection and constructive self-criticism. Inspired by scholar Cida Bento^14^, I propose that everyone look inward and analyze how the pact of whiteness manifests in the scientific milieu. First, we epidemiologists (especially white people) must reflect on the privileges and opportunities that have been afforded to us. We must also recognize the importance of other knowledges, epistemologies, and analytical methods in knowledge production. Other ways of doing science!

Cida Bento denounces and questions whiteness and its harmful consequences for social relations in Brazil. Through this silent pact, white people’s privileges are perpetuated to maintain the status quo^14^. How has this occurred in collective health, especially in the everyday practices of epidemiology? The issue of the narcissistic pact of whiteness is a pact that is silent (not necessarily verbalized) but attention must be paid to the details in debates, events, and scientific publications. In the dossier on the health of the Black population, organized by members of Abrasco’s Working Group on Racism and Health, Rosana Onocko, in a review, calls on white people to reflect: *what strategies and actions would we, as white people, be capable of contributing to dismantle this pact of whiteness?* ^15^

What epidemiological studies exist on the health of the white population, and what discussion of whiteness do we have in our field? Generally, in teaching, research, outreach, and technical cooperation, our actions are directed at looking at “the other.” Considering also that white, Indigenous, Asian, pardo, and Black people are all racialized, there is unfortunately a difficulty within whiteness in not seeing itself as universal, the gold standard or reference in analyses, because it shows better health and longevity indicators.

Scientific publications addressing ethnic-racial health inequities are still insufficient. This reflects the “pact of whiteness” and racism. There is an urgent need to expand scientific production and provide visibility, with scientific evidence, to the injustices affecting this population’s lives, as well as to direct actions aimed at health equity. Increasing affirmative-action measures for undergraduate and graduate students in our programs is not enough; these students often cannot advance in their careers. Funding Black researchers and Black-led research through grants, programs, and support initiatives with affirmative-action criteria for race, gender, and generation is essential to carry out studies and complete projects that will help improve public policies^16^.

Another essential measure to improve scientific production is expanding Black women’s and men’s leadership in large national and international multicenter projects. Additionally, greater listening and collective work with members of Black communities, and according to the worldview of Afro-Brazilian traditional communities, such as members of Afro-Brazilian religions and quilombolas, is fundamental*.

How can we ensure more equitable funding and carry out studies that use mixed methods and encompass evidence produced in both qualitative and quantitative fields? To deepen the issue of racism in health, it is necessary to engage with research groups in the social sciences of health and with popular masters and community experts, thereby broadening the theoretical and rational spectrum that epidemiology alone cannot fully address. Restricting the debate to the health sector is not enough to deepen these issues. Epidemiologists need to strengthen partnerships and learn from other sectors of society, starting with social movements and civil society organizations. In our field, we do not value and often make invisible the knowledge that comes from Afro-Brazilian and Indigenous worldviews; we also contrast and challenge the cis-heteronormative conception^17^.

There is also a need to more fully incorporate different languages and modes of expression, such as art, literature, and audiovisual communication, to convey epidemiological study results that are not always easy for the people most affected by the health issues to understand. If we restrict ourselves to articles published in high-impact international journals that charge fees inaccessible to most scholars outside research groups, the scope of efforts to confront racism in epidemiology will remain limited.

Brasil and Trad^18^ investigated how people from different social movements that shape the health agenda for the Black population view the potential of epidemiology as a management tool. For Black people living in communities and organized social movements, epidemiological data that generate information and knowledge are weapons that can be used to plan public policies to confront racism.

The inclusion of “dissident” or non-hegemonic bodies in graduate and undergraduate programs in collective health has prompted reflections on the structuring processes of domination within the spaces, languages, and logics that make up those programs and curricula. It is this student body that daily challenges us toward change and advances in intersectional and decolonial debate in epidemiology^19,20^.

In an activity of the curricular component Epistemology and Methodology in Health, a group of Black women doctoral students from the Graduate Program in Collective Health at the Institute of Collective Health of the Federal University of Bahia (ISC/UFBA) wrote:

*“As scientists, we advocate for a science that not only challenges the maintenance of established powers but also positions itself as* an agent of liberation, escaping hegemonic narratives. Our vision is of a science committed to solving problems that affect all populations, with special attention to those most vulnerable. We seek a science that recognizes and values legitimate knowledges that for too long have been diminished, erased, or usurped.”

During the “12th Brazilian Congress of Epidemiology: Epidemiology and the Complexity of Health Challenges”, we had the opportunity to reflect, learn, and act on the various issues crossing Brazil’s population health, such as social inequalities and contemporary problems. Racism and all its manifestations are an old, complex problem that we have not yet fully confronted. I heard Abrasco members speak about the need for radicalism in the struggle for democracy and social justice. That struggle requires a radical individual and collective stance in confronting racism. How long will we, as public health professionals, continue to collude in maintaining and updating racism through our practices?

All topics discussed at the congress, such as digital health, the climate crisis, mental health, etc., must be problematized and examined through the lenses of racism, sexism, ableism, ageism, and other forms of discrimination. We need to recognize these gaps in order to advance, as our 5th Master Plan for the Development of Epidemiology in Brazil indicates.

* Elaborated by: Carol Cardoso Rodrigues; Lívia Ferreira Reis; Mara Viana Cardoso Amaral; Nubia dos Reis Pinto; Olbichoo Lexius; Samantha Vitena Barbosa and Silvana Oliveira da Silva.
