# Supplementary material for: Uncovering inequities: the role of epidemiology in confronting racism
Source: Rev Bras Epidemiol. 2026 Jul 24;28(Suppl 1):e250008supl1. doi: 10.1590/1980-549720250008.supl.1 (PMC13405550; doi:10.1590/1980-549720250008.supl.1)
Supplement: Translation

# Translation: Desvelando iniquidades: o papel da epidemiologia no enfrentamento do racismo

**DOI:** 10.1590/1980-549720250008.supl.1.2

**Authors:** Joilda Silva Nery

**Affiliations:** I Universidade Federal da Bahia, Instituto de Saúde Coletiva – Salvador (BA), Brasil.

**Keywords:** Racismo, Epidemiologia, Saúde da população negra, Desigualdades raciais, Saúde coletiva

## Abstract

O presente artigo discute o papel da epidemiologia e da saúde coletiva no enfrentamento do racismo e das desigualdades raciais em saúde no Brasil. As reflexões aqui apresentadas foram inspiradas na conferência “Desvelando iniquidades: o papel da epidemiologia no enfrentamento do racismo”, ocorrida no 12o Congresso Brasileiro de Epidemiologia, em 2024. Por meio de uma análise crítica e histórica, busca-se compreender como a epidemiologia brasileira — consolidada na América Latina e reconhecida por suas contribuições à saúde coletiva — ainda reproduz mecanismos de invisibilização de pesquisadoras e pesquisadores negros e indígenas. Argumenta-se que o combate ao racismo não pode ser tarefa isolada de um grupo específico, mas um compromisso coletivo *e* ético que envolva toda a comunidade científica. Os dados de morbimortalidade falam por si e pela maioria da população brasileira. É preciso debater de forma mais explícita o racismo na área de epidemiologia e refletir como as nossas instituições e grupos se organizam e operam de acordo com as relações de poder. Além disso, propõe-se que temas contemporâneos, como saúde digital, mudanças climáticas e saúde mental, sejam analisados sob perspectivas interseccionais, considerando raça, gênero, classe e outras dimensões de desigualdade. O texto enfatiza que o reconhecimento do racismo como determinante social da saúde é condição essencial para a construção de uma epidemiologia comprometida com a equidade e com o direito à vida. É necessário reconhecer essas lacunas para avançar, conforme aponta o nosso V Plano Diretor para o Desenvolvimento da Epidemiologia no Brasil.

Essa escrita é oriunda das reflexões compartilhadas na conferência intitulada “Desvelando iniquidades: o papel da epidemiologia no enfrentamento do racismo”, que ocorreu no 12° Congresso Brasileiro de Epidemiologia, realizado em 2024, e abordou o papel da epidemiologia no enfrentamento das desigualdades raciais em saúde no Brasil^1^. O texto propõe uma análise crítica sobre o compromisso ético e político da epidemiologia diante das iniquidades raciais em saúde, problematizando a forma como a área se estrutura, produz conhecimento e se relaciona com as populações afetadas pelo racismo.

Essa trajetória tem sido fundamental para compreender como a diversidade de “saberes e fazeres” pode fortalecer o conhecimento epidemiológico produzido nas universidades e instituições de pesquisa. Para tanto, devemos primeiramente questionar com quem, para quem e como dialogamos na área. Como a saúde coletiva e a epidemiologia podem contribuir para o enfrentamento efetivo do racismo? Isso requer um comprometimento genuíno da maioria das pessoas que compõem a área. Muitos encontros, convergências, desapontamentos, afetos, desafetos, aprendizados e trocas entre pares me fazem refletir sobre a nossa responsabilidade e o nosso engajamento como epidemiologistas nessa agenda política.

A epidemiologia latino-americana e brasileira é um campo consolidado, mas, a despeito da sua inconteste contribuição para a saúde da população, é marcada pela invisibilização de pesquisadoras e pesquisadores negros que contribuíram com essa história. Comumente, quando a história da epidemiologia é contada, não são considerados os movimentos das mulheres negras que atuaram no âmbito da Reforma Sanitária Brasileira. Autoras como Alaerte Coutinho, Maria Inês Barbosa, Edna Araújo, Denize Ribeiro, Lúcia Xavier, Jurema Werneck e Fernanda Lopes contribuíram para situar o racismo como determinante social da saúde e para reivindicar a inserção do tema nas agendas acadêmicas e políticas. Entretanto, suas contribuições ainda são pouco reconhecidas nos currículos e na historiografia da epidemiologia brasileira. Fátima de Oliveira, em sua contribuição na tese de Sueli Carneiro^2^, nos convoca a pensar que invisibilizar é uma velha e vitoriosa estratégia política racista.

Como nos alerta Chimamanda Ngozi Adichie^3^, é o perigo da história única contada por quem está em posição de privilégio no campo, pois muitas intelectuais negras e negros não aparecem. Como nos ensinam as nossas lideranças mais velhas, temos história, memória, e precisamos preservá-las. Nesse texto, eu dialogo com intelectuais de diferentes regiões, formações e gerações que têm contribuído diuturnamente e ao longo de décadas no enfrentamento do racismo por meio da produção de evidências de como as iniquidades étnico-raciais afetam o nascer, adoecer e morrer^4^ das pessoas negras e indígenas da América Latina e do Brasil de modo desigual. Essas pessoas têm dado uma enorme contribuição para a saúde coletiva e epidemiologia, devendo ser citadas e consideradas como referências.

A despeito dos avanços conquistados com muita luta, nós, pessoas pretas, pardas e indígenas, queremos e podemos avançar muito mais no fortalecimento da saúde coletiva. Dados sociodemográficos e epidemiológicos oriundos de diferentes fontes apontam para as piores condições de vida e saúde da população negra no mundo e no Brasil. Parte dos dados necessários para fazer as análises de situação de saúde está disponível nos artigos, capítulos, painéis informativos e debates. Peço licença para falar por milhares de pessoas que compõem os dados que são analisados e discutidos nas aulas, pesquisas, e no apoio à tomada de decisão no Sistema Único de Saúde (SUS).

A população negra (composta por pessoas pardas e pretas no Brasil) não é homogênea e representa parcela significativa das comunidades tradicionais, quilombolas, ribeirinhas; de pescadores artesanais; dos que vivem em situação de rua; das pessoas privadas de liberdade; das que vivem na extrema pobreza e em domicílios que não respondem aos padrões de habitabilidade, que não contam com abastecimento de água e/ou esgotamento sanitário como nas favelas; daqueles que apresentam menores rendimentos ou sobrevivem da informalidade; dos que dependem do lixo de natureza reciclável ou não; das empregadas domésticas; das pessoas idosas; dos que estão em situação de insegurança alimentar; dos que têm dificuldades de acesso a serviços e equipamentos de saúde, assistência social e educação^5,6^. Estamos falando de pessoas que vivenciam o racismo e diferentes manifestações da violência no seu cotidiano, com repercussões importantes nas subjetividades, na saúde mental e física. Como não se indignar?

O lugar que as pessoas ocupam na sociedade resulta de processos históricos e das condições políticas, econômicas e sociais que moldam as desigualdades produzidas segundo raça, gênero, geração e classe social. No que se refere às relações raciais, é importante lembrar que o termo “raça” foi, por longo período, utilizado para hierarquizar populações, sustentando concepções de supremacia racial e de pureza biológica. Conforme argumentam Lopes e Werneck, o conceito de raça não se constitui de forma neutra: ele se estrutura a partir do próprio racismo, entendido como um mecanismo social de exclusão que afeta a todos, embora de maneira diferenciada conforme a posição que cada grupo ocupa na hierarquia social^7^.

O diálogo com colegas e as leituras dos textos de diferentes áreas do conhecimento reforçam que a epidemiologia tem um papel fundamental ao evidenciar as disparidades sociais e raciais de saúde, mas que é preciso avançar mais. A epidemiologia é uma área da saúde coletiva pautada na produção de evidências, orientadora da tomada de decisões em saúde e deve considerar o enfrentamento às iniquidades raciais como estratégia prioritária para a melhoria dos indicadores de morbimortalidade no Brasil; assumir o racismo como central no processo de determinação social das desigualdades em saúde. Com racismo, não há saúde^7^, e a epidemiologia deve apontar prioridades e caminhos para superação das iniquidades.

O racismo, enquanto fenômeno ideológico e estruturante, compreende um conjunto de atitudes baseadas em preconceitos raciais, comportamentos discriminatórios, mecanismos institucionais e disposições sociais que atribuem valores negativos à diversidade e produzem significados hierarquizados entre grupos sociais. Essas dinâmicas sustentam padrões de exclusão que resultam em desigualdades raciais persistentes. O racismo, portanto, orienta as formas de convivência, regula as relações sociais e influencia a organização e o funcionamento das instituições. No caso da população negra, as desigualdades nos processos de nascer, viver, adoecer e morrer não são naturais nem inevitáveis — são produto de um sistema social que poderia ser transformado caso o racismo fosse amplamente reconhecido como determinante social da saúde e seu enfrentamento assumido como prioridade ética e política dentro e fora dos serviços que compõem o SUS^4,8,9^.

Como pesquisadoras e pesquisadores epidemiologistas não negros observam as pessoas pardas, pretas e indígenas que compõem as “amostras” dos estudos? Diversos estudos epidemiológicos publicados apresentam o destaque “recorte de raça”, “recorte de gênero” (muitas vezes pela variável sexo categorizada em masculino e feminino), “recorte de raça/gênero”. Essa perspectiva, muitas vezes, é sedimentada por homens brancos que não se percebem enquanto integrantes de um grupo que também é racializado, mas que não sofre racismo. Com isso, fazem análises epidemiológicas com recorte de raça ao se referir à população parda, preta e indígena. De acordo com o último censo do Instituto Brasileiro de Geografia e Estatística (IBGE)^10^, a população negra (pardos e pretos) representa 55,5%, ou seja, a maioria da população brasileira. Nesse sentido, é um equívoco conceitual considerar estudos que discutem a questão racial como “recorte”.

Doenças crônicas, infecciosas; acidentes relacionados ao trabalho; ocupações perigosas e/ou insalubres, como trabalhadoras de serviços gerais, limpeza, agricultura, construção civil etc. Quais são os grupos étnico-raciais mais acometidos ou que morrem mais por essas doenças/acidentes? De quais raça/cor, gênero e faixa etária são a maioria desses trabalhadores e trabalhadoras? Nesse sentido, também se torna necessário o maior detalhamento étnico-racial de doenças/agravos relacionados ao trabalho no Brasil^11^.

Os diálogos com colegas da área de saúde coletiva apontam que o racismo também é reiteradamente praticado por atrizes e atores políticos que atuam nas instituições e nos campos mais progressistas, que constroem as narrativas na área da saúde e da epidemiologia, mas que ainda assim se negam a compreender a sua profundidade como estratégia de adoecimento e morte que vitimiza a população negra e indígena. As discussões apresentadas nos trabalhos acadêmicos e eventos da área são pautadas majoritariamente em desigualdades de classe; os dados sobre raça/cor são coletados, apresentados e discutidos sem o aprofundamento de como o racismo é um determinante social da saúde fundamental na compreensão das exposições e dos desfechos investigados. A lacuna da maioria dos estudos epidemiológicos é desconsiderar que a variável sociodemográfica raça/cor é atravessada pelo racismo e que este deve estar presente na leitura feita sobre as desigualdades que essa variável desvela. Desse modo, o enfrentamento do racismo por meio de pesquisas, ensino e extensão na epidemiologia não deve se restringir às poucas pesquisadoras negras, negros e indígenas e que enfrentam as diferentes dimensões do racismo cotidianamente. É preciso debater de forma mais explícita o racismo na área de epidemiologia e fazer uma reflexão de como as nossas instituições, grupos de pesquisa e coletivos temáticos se organizam e operam nas suas relações de poder.

A questão racial no Brasil não é pauta identitária. É pauta tão estruturante e transversal quanto as desigualdades de classe e, pelo processo de formação histórica do nosso país, não deve ser desconsiderada. Os dados falam por si e pela maioria da população brasileira. As discussões sobre as diretrizes do V Plano Diretor para o Desenvolvimento da Epidemiologia, da Associação Brasileira de Saúde Coletiva (Abrasco)^12^, consideraram a questão das desigualdades/iniquidades em saúde, especialmente no que se refere às populações mais afetadas pelas doenças e agravos; porém, ainda não enfrentam de modo transversal a questão racial, privilegiando as “desigualdades sociais”. Embora as bases teóricas sobre as desigualdades de classe sejam necessárias e importantes, nem sempre consideram de forma adequada as discriminações relacionadas a raça, etnia, gênero e geração, destacando a necessidade de ampliar o olhar sobre as questões estruturais que afetam a saúde da população. O ponto de partida para uma epidemiologia que reflita a diversidade do Brasil reside na compreensão genuína de que cada dado quantitativo representa vidas e histórias.

No âmbito da vigilância em saúde, a discussão étnico-racial com enfoque na população negra e indígena ainda é incipiente no Brasil. Frequentemente, a variável raça/cor é tratada apenas como mais um item dentro de um conjunto de diversas variáveis sociodemográficas. A maior parte das publicações sobre doenças infecciosas que afetam a população negra resulta de pesquisas qualitativas, enquanto os poucos estudos epidemiológicos existentes ainda não aprofundam a análise do racismo como fator estruturante na determinação social do processo saúde-doença. Grande parte das discussões sobre enfermidades infectocontagiosas, tanto na epidemiologia quanto na prática clínica, concentra-se nas doenças em si, invisibilizando as pessoas acometidas e com os piores desfechos do tratamento, quando este é acessível^11^.

Repetir de forma imparcial e acrítica esses dados epidemiológicos, sem aprofundar os motivos subjacentes, causa um desconforto semelhante ao das aulas de história do Brasil nas escolas. Nelas, as trajetórias das populações indígenas e negras eram apresentadas exclusivamente sob a ótica da invisibilização e da escravização. Coletar, analisar e sistematizar dados epidemiológicos que mostram que a população negra apresenta os piores indicadores de morbimortalidade, situando o debate apenas nas desigualdades de classe, constitui uma forma de narrar a história das “outras e outros”^3^.

Nas instituições de ensino e/ou pesquisa que possuem a área de epidemiologia, frequentemente as interseccionalidades entre raça, etnia, gênero e geração ainda continuam sistematicamente negligenciadas. Apesar do crescimento no número de publicações que utilizam o quesito raça/cor, ainda são escassos os estudos epidemiológicos com aprofundamento das discussões teóricas e metodológicas sobre os impactos do racismo na saúde com base em uma lente interseccional. Nesse cenário, remeto à Câmara Phyllis Jones, que nos alerta sobre o racismo institucionalizado como uma inação evidente diante da necessidade^13^. Temos a necessidade de extrapolar a discussão epidemiológica para as desigualdades étnico-raciais, mas a omissão ao fazê-lo contribui para a manutenção e perpetuação do racismo.

O racismo faz parte do processo de determinação social da saúde, e a dimensão institucional afeta as relações de poder nos grupos e instituições e os modos de produção dos dados, informação e conhecimento na área de epidemiologia. Primeiro precisamos fazer um exercício de reflexão e autocrítica construtiva. Inspirados na intelectual Cida Bento^14^, proponho que cada um/uma olhe para si e analise como o pacto da branquitude se manifesta no meio científico. Primeiro, nós, epidemiologistas, sobretudo as pessoas brancas, precisamos refletir sobre os privilégios e oportunidades que nos foram garantidos. Além disso, reconhecer a importância dos demais saberes, epistemologias e métodos analíticos na produção de saberes. Outras formas de fazer ciência!

Cida Bento denuncia e questiona a branquitude e suas consequências nocivas para as relações sociais no Brasil. Por meio desse pacto silencioso, perpetuam-se privilégios de pessoas brancas para se manter no status quo^14^. Como isso tem ocorrido na saúde coletiva, especialmente nas práticas cotidianas da área de epidemiologia? A questão do pacto narcísico da branquitude é um pacto que é silencioso, não necessariamente é verbalizado; mas é preciso ter atenção aos detalhes nos debates, eventos e publicações científicas. No dossiê sobre saúde da população negra, organizado por membros do Grupo de Trabalho (GT) Racismo e Saúde da Abrasco, Rosana Onocko, por meio de uma resenha, convoca as pessoas brancas a refletirem: *quais estratégias e ações seríamos os brancos capazes de contribuir para a demolição desse pacto de branquitude?* ^15^

Quais são os estudos epidemiológicos sobre saúde da população branca e qual a discussão sobre branquitude fazemos na nossa área? Geralmente, no ensino, na pesquisa, na extensão e cooperação técnica, nossas ações se direcionam para olhar o “outro”. Considerando também que as pessoas brancas, indígenas, amarelas, pardas e pretas são todas racializadas, mas, infelizmente, há uma dificuldade da branquitude em não se perceber como universal, padrão-ouro, referência nas análises por apresentarem melhores indicadores de saúde e longevidade.

As publicações científicas que abordam as iniquidades étnico-raciais em saúde ainda não são suficientes. Isso é um reflexo do “pacto da branquitude” e do racismo. É urgente a necessidade de ampliar a produção científica e dar visibilidade, com evidências científicas, às injustiças que impactam a vida dessa população, bem como direcionar ações que objetivam a equidade em saúde. Não basta o incremento das ações afirmativas com estudantes de graduação e pós-graduação nos nossos cursos, sendo a base dos nossos grupos; muitas vezes, esses estudantes não conseguem ascender na carreira. O financiamento de pesquisadores e pesquisadoras negros e negras e da pesquisa, com editais, programas e iniciativas de fomento com ações afirmativas considerando raça, gênero e geração, é fundamental para realizar os estudos e concluir os projetos que vão contribuir para aprimorar as políticas públicas^16^.

Uma outra medida muito necessária nesse esforço para melhorar a produção científica é a ampliação da liderança de mulheres e homens negros em grandes projetos multicêntricos nacionais e internacionais. Além disso, é fundamental uma maior escuta e construção coletiva com membros de comunidades negras e segundo a cosmovisão de comunidades tradicionais afro-brasileiras, a exemplo de membros de religiões afro-brasileiras e quilombolas*.

Como considerar o financiamento mais equitativo e a realização de estudos que considerem métodos mistos e abarquem evidências produzidas nas áreas qualitativas e quantitativas? Para aprofundar o tema do racismo em saúde, é necessário dialogar com grupos de pesquisa da área de Ciências Sociais em Saúde e mestras/mestres populares, conseguindo ampliar o espectro racional teórico que somente na epidemiologia eu não conseguiria avançar. Restringir o debate apenas ao setor saúde não é suficiente para aprofundar essas questões. Os epidemiologistas precisam fortalecer as parcerias e aprender com outros setores da sociedade, a começar pelos movimentos e organizações da sociedade civil. Na nossa área, não valorizamos e invisibilizamos os saberes oriundos das cosmovisões afro-brasileiras e indígenas; ainda, contrapomos a concepção cis-heteronormativa^17^.

Além disso, há uma necessidade de maior incorporação de diferentes linguagens, como arte, literatura e comunicação audiovisual, para explicar os resultados dos estudos epidemiológicos, que nem sempre são de fácil compreensão para as pessoas mais interessadas nos problemas de saúde discutidos. Se nos restringirmos aos artigos publicados nas revistas internacionais, com alto fator de impacto e que cobram taxas inacessíveis para a maioria das intelectuais que não estão nos grupos de pesquisa, o leque de atuação do enfrentamento do racismo na epidemiologia continuará limitado.

Brasil e Trad^18^ investigaram como as pessoas de diferentes movimentos sociais que pautam a saúde da população negra consideram o potencial da epidemiologia como instrumento de gestão. Para as pessoas negras que estão nos territórios e movimentos sociais organizados, os dados epidemiológicos que geram informação e conhecimento são armas que podem ser utilizadas no planejamento de políticas públicas de enfrentamento do racismo.

A entrada de corpos “dissidentes” ou não-hegemônicos nos programas de pós-graduação e graduação em saúde coletiva tem gerado reflexões sobre processos de dominação estruturantes dos espaços, linguagens e lógicas que compõem tais programas e estruturas curriculares. É esse corpo discente que cotidianamente nos provoca em direção a mudanças e avanços no debate interseccional e contracolonial na epidemiologia^19,20^.

Em uma atividade do componente curricular de Epistemologia e Metodologia em Saúde, um grupo de mulheres negras doutorandas do Programa de Pós-Graduação em Saúde Coletiva do Instituto de Saúde Coletiva da Universidade Federal da Bahia (ISC/UFBA) escreveu:

*“Enquanto cientistas, defendemos uma ciência que não apenas desafie a manutenção de poderes estabelecidos, mas que também se posicione como um* agente de libertação, escapando das narrativas hegemônicas. Nossa visão é de uma ciência que se compromete com a resolução de problemas que afetam todas as populações, com especial atenção para aquelas mais vulnerabilizadas. Buscamos uma ciência que reconheça e valorize saberes legítimos que por muito tempo foram diminuídos, anulados ou usurpados.”

Durante o “12° Congresso Brasileiro de Epidemiologia: a epidemiologia e a complexidade dos desafios sanitários”, tivemos a oportunidade de refletir, aprender e atuar sobre as diversas questões que atravessam a saúde da população brasileira, como as desigualdades sociais e os problemas contemporâneos. O racismo e todas as suas manifestações são um problema antigo, complexo e que ainda não enfrentamos na sua totalidade. Eu ouvi de “abrasquianos” sobre a necessidade da radicalidade na luta pela democracia e justiça social. Essa luta perpassa uma postura individual e coletiva radical no enfrentamento do racismo. Até quando nós, sanitaristas, vamos compactuar com a manutenção e atualização do racismo por meio das nossas práticas?

Todos os temas discutidos no congresso, como saúde digital, crise climática, saúde mental etc., devem ser problematizados e lidos sob as lentes do racismo, sexismo, capacitismo, etarismo e das distintas formas de discriminação. Precisamos reconhecer essas lacunas para avançar, conforme aponta o nosso V Plano Diretor para o Desenvolvimento da Epidemiologia no Brasil.

* Elaborado por: Carol Cardoso Rodrigues; Lívia Ferreira Reis; Mara Viana Cardoso Amaral; Nubia dos Reis Pinto; Olbichoo Lexius; Samantha Vitena Barbosa e Silvana Oliveira da Silva.

## DECLARAÇÃO DE IA E TECNOLOGIAS ASSISTIDAS POR IA NO PROCESSO DE ESCRITA:

Durante a preparação deste texto, a autora utilizou o Chat GPT em outubro de 2025 para avaliação do texto melhorias gramaticais e semânticas ao texto. Após o uso destas ferramentas, a autora revisou e editou o conteúdo em conformidade com o método científico e assume total responsabilidade pelo conteúdo da publicação.
